# The Brain Is the Rate-Limiting Organ of Longevity: A Brain-First Systems Framework for Aging

**DOI:** 10.7759/cureus.101106

**Published:** 2026-01-08

**Authors:** Shaheen E Lakhan

**Affiliations:** 1 Bioscience, Boricua Bio, San Juan, USA; 2 Bioscience, Global Neuroscience Initiative Foundation, Miami, USA; 3 Neurology, Western University of Health Sciences, Pomona, USA; 4 Neurology, A.T. Still University School of Osteopathic Medicine in Arizona, Mesa, USA; 5 Medicine, Morehouse School of Medicine, Atlanta, USA

**Keywords:** brain aging, central nervous system, cognitive resilience, healthspan, longevity medicine, neuro-longevity, systems neuroscience

## Abstract

Longevity research has traditionally emphasized peripheral organ systems, metabolic optimization, and molecular aging pathways, while comparatively neglecting the central nervous system as the primary determinant of healthspan. This editorial advances the thesis that the brain functions as the rate-limiting organ of longevity. Drawing on systems neuroscience, clinical neurology, and evidence from neuropsychiatric and neurodegenerative disease, it is argued that progressive disruption of neural networks governs functional decline across multiple physiological systems, regardless of peripheral biological age. Cognitive resilience, autonomic regulation, sleep integrity, affective stability, and behavioral capacity are centrally mediated processes that determine an individual’s ability to maintain homeostasis over time. When brain function deteriorates, lifespan may persist, but meaningful healthspan collapses. A Brain-First Longevity Framework (BFLF) is proposed that prioritizes preservation and restoration of neural network function as foundational to extending durable, functional longevity. BFLF has direct implications for clinical practice, therapeutic development, and the future architecture of longevity medicine.

## Editorial

Introduction

The pursuit of longevity has become one of the most prominent objectives of modern medicine, biotechnology, and public health. Advances in cardiovascular care, oncology, infectious disease, and metabolic management have succeeded in extending lifespan across populations. However, these gains have exposed a growing disconnect between survival and function. Increasingly, individuals live longer while experiencing progressive cognitive decline, emotional dysregulation, sleep disruption, and loss of independence. This phenomenon reflects a fundamental limitation in prevailing longevity paradigms.

Most longevity strategies conceptualize aging as a problem of peripheral organ deterioration or molecular damage. Interventions are therefore directed toward optimizing metabolic pathways, reducing cardiovascular risk, slowing cellular senescence, or correcting biochemical abnormalities. While these approaches address important contributors to mortality, they insufficiently account for the central nervous system, which governs the coordination, regulation, and adaptive capacity of all other physiological systems.

The brain is not merely another organ subject to aging. It is the system that integrates internal and external information, orchestrates physiological responses, and enables behavioral adaptation over time. When brain function declines, the capacity to maintain health across organ systems diminishes, even when those organs remain structurally intact, a relationship supported by evidence that neuroimaging-derived brain age predicts mortality independently of peripheral risk factors [1]. Longevity efforts that do not explicitly prioritize brain integrity, therefore, fail to address the primary determinant of sustained healthspan.

In longevity medicine, a rate-limiting organ is defined as the biological system that most constrains sustained functional adaptation over time, regardless of the preservation of peripheral organ structure or molecular integrity [2]. By this definition, the central nervous system, not any peripheral organ, constitutes the rate-limiting organ of longevity.

**Figure 1:**
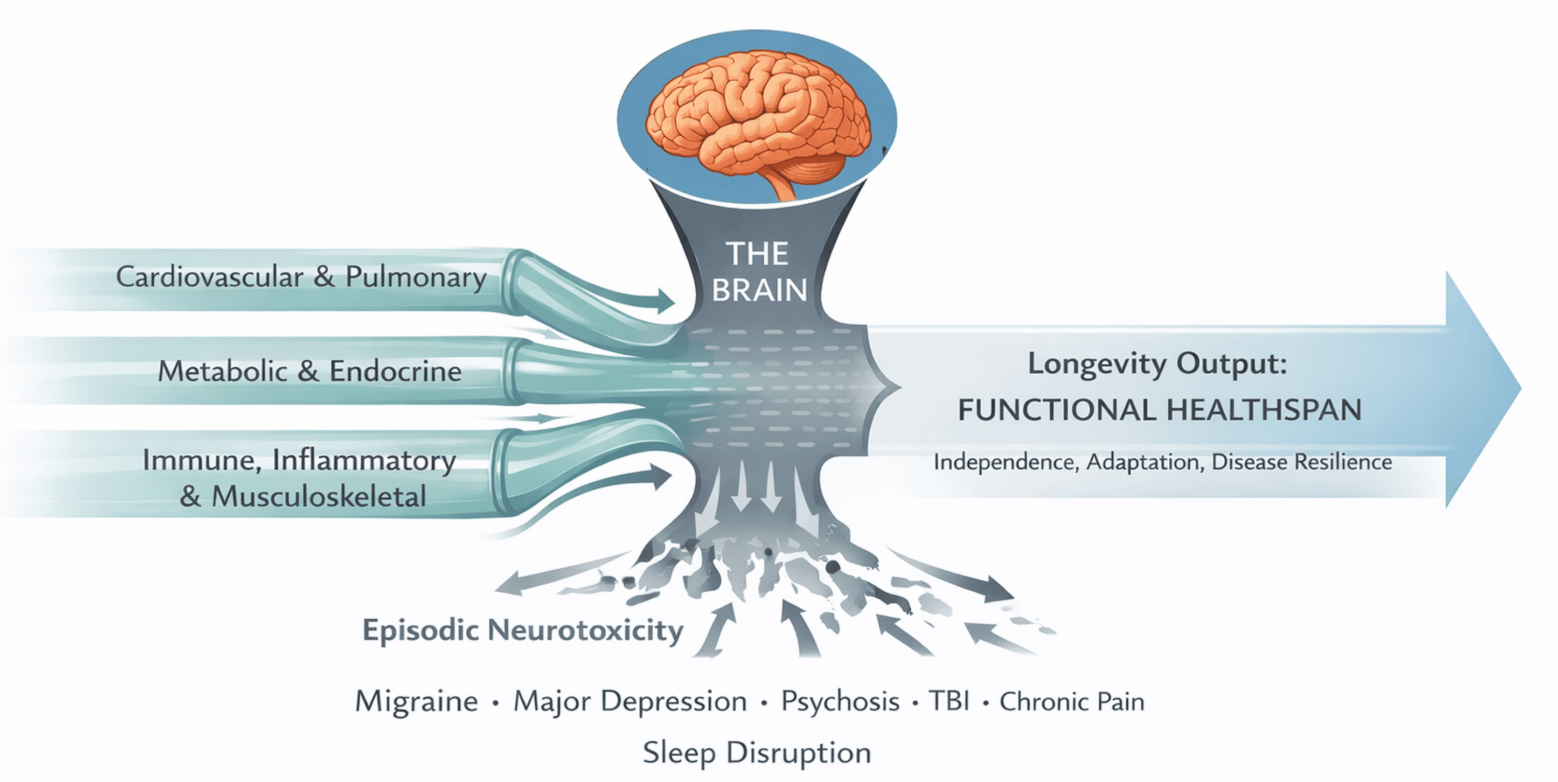
The brain as the rate-limiting organ of longevity This figure illustrates the central nervous system as the rate-limiting constraint on functional healthspan. Peripheral physiological systems, including cardiovascular, metabolic, endocrine, immune, inflammatory, and musculoskeletal domains, converge toward a common longevity output. The brain functions as a biological bottleneck, such that overall healthspan is constrained by neural network integrity, cognitive resilience, autonomic regulation, sleep architecture, and emotional regulation, regardless of peripheral system optimization. Episodic neurotoxic events, including migraine, major depressive disorder, psychosis, traumatic brain injury, chronic pain, and sleep disruption, further constrict this bottleneck by accelerating loss of neural network efficiency. As a result, longevity output, defined as sustained functional healthspan characterized by independence, adaptive capacity, and disease resilience, is fundamentally limited by brain function rather than by peripheral organ preservation alone. Image Credit: Author

This editorial advances a systems neuroscience thesis that reframes longevity as being constrained not by the slowest peripheral organ to fail, but by the central system that limits adaptive capacity. Recognizing the brain in this role necessitates a fundamental shift in how longevity is defined, measured, and therapeutically pursued. This systems-level view of longevity, in which brain function constrains downstream physiological aging, is illustrated in Figure 1.

The concept of a rate-limiting organ in aging

In engineering and systems biology, a rate-limiting element constrains the performance of an entire system regardless of the capacity of downstream components. Enhancing non-limiting elements yields a marginal benefit if the primary constraint remains unaddressed. Aging, as a complex biological process, follows the same principle.

Traditional aging models implicitly treat organ systems as semi-independent contributors to mortality. While failure of organs such as the heart, kidneys, or liver can directly cause death, their long-term function is inseparable from central neural regulation. Autonomic output governs cardiovascular tone and rhythm. Neuroendocrine signaling modulates metabolism, immune activity, and stress responses. Circadian and sleep-wake regulation influences nearly every aspect of systemic physiology [3]. Behavioral decisions, including medication adherence, nutrition, physical activity, and social engagement, are entirely brain-dependent.

From a systems perspective, peripheral organ decline frequently reflects downstream consequences of central dysregulation rather than isolated primary failure. The organ that most limits sustained adaptation, resilience, and coordination across time, therefore, functions as the rate-limiting organ of longevity. By this definition, the brain occupies a unique and dominant position. While failure of peripheral organs such as the heart or kidneys may represent the proximate cause of death, progressive neural dysfunction determines the trajectory of physiological decline by eroding homeostatic control, stress tolerance, and adaptive capacity long before terminal organ failure occurs.

The brain as the master regulator of physiological aging

The brain maintains physiological stability through continuous integration of sensory input, internal state monitoring, and predictive control. Autonomic networks regulate heart rate variability, vascular tone, and blood pressure stability, all of which are associated with cardiovascular outcomes and mortality risk. Hypothalamic circuits coordinate endocrine signaling governing glucose metabolism, appetite, thermoregulation, and inflammatory balance. Limbic and cortical networks modulate stress reactivity, emotional regulation, and immune responses.

Sleep architecture, another centrally governed process, plays a critical role in metabolic regulation, synaptic homeostasis, and glymphatic clearance of neurotoxic metabolites [4]. Disruption of sleep, whether through aging or disease, accelerates cognitive decline and systemic dysfunction. Executive and motivational systems further determine engagement with preventive care, rehabilitation, and social interaction, all of which strongly influence health outcomes.

Importantly, these regulatory functions decline with age even in the absence of overt neurodegenerative disease. Subclinical cognitive impairment, sleep fragmentation, autonomic instability, and affective dysregulation frequently precede measurable peripheral organ failure. In aging, what fails first is not strength, endurance, or organ reserve, but neural coordination. These early failures reduce the organism’s capacity to adapt to stressors, increasing vulnerability to illness and accelerating functional decline.

Longevity is therefore constrained not simply by the durability of peripheral tissues, but by the brain’s ability to sustain coherent regulation and integration across physiological systems. At a mechanistic level, this central regulatory role is mediated through integrated neuro-endocrine-immune signaling. Hypothalamic circuits function as an aging pacemaker by coordinating autonomic output, neuroendocrine signaling via the hypothalamic-pituitary-adrenal axis, and immune modulation [5]. Age-related dysregulation within these circuits amplifies systemic inflammation, metabolic instability, and stress vulnerability, accelerating functional decline across peripheral organs. From this perspective, molecular and organ-level aging processes are not independent phenomena, but downstream expressions of progressive central regulatory failure

Neural network efficiency as the biological currency of longevity

At a mechanistic level, brain aging can be understood as a progressive reduction in neural network efficiency. Efficient neural networks maintain high signal-to-noise ratios, enabling accurate information processing, adaptive learning, and coordinated physiological regulation. Aging, disease, and recurrent neural stressors degrade this efficiency, leading to increased noise, impaired synchronization, and reduced adaptability.

Episodic neurotoxic events accelerate this process by imposing transient but cumulative disruptions to network integrity. Loss of function often reflects degraded network performance rather than irreversible neuronal loss, consistent with evidence that increased brain age gap predicts cognitive decline, neuropsychiatric morbidity, and mortality across populations [6]. From this perspective, cognitive decline, emotional dysregulation, and sleep fragmentation are manifestations of declining network efficiency rather than isolated symptoms.

Longevity, therefore, depends not solely on preserving neural tissue, but on sustaining efficient information processing across distributed brain networks over time. Neural network efficiency emerges as a central biological currency of durable healthspan.

Episodic neurotoxicity and nonlinear brain aging

Aging of the brain is not purely a gradual, time-dependent process. Episodic neurological and neuropsychiatric events can accelerate neural aging through cumulative network disruption. Conditions such as migraine, major depressive disorder, psychotic illness, traumatic brain injury, chronic pain syndromes, and sleep disorders impose recurrent physiological and metabolic stress on neural circuits. Although these episodes may remit clinically, they often leave residual alterations in network efficiency and connectivity.

A unifying mechanism underlying these diverse episodic conditions is neuroinflammation [7]. Recurrent activation of inflammatory signaling within the central nervous system disrupts synaptic efficiency, impairs network synchronization, and increases neural metabolic burden. While individual episodes may appear transient, their cumulative inflammatory impact progressively degrades neural network performance. This provides a biological explanation for how episodic neurological or psychiatric events occurring decades earlier can meaningfully constrain cognitive resilience and healthspan later in life

Each episode contributes to increased allostatic load within the central nervous system [8]. Over time, the brain’s capacity for efficient information processing, emotional regulation, and autonomic control diminishes. This process can unfold silently for years or decades, manifesting as reduced cognitive flexibility, increased stress sensitivity, and impaired sleep long before classical neurodegenerative diagnoses emerge.

This episodic model of brain aging explains why individuals with recurrent neuropsychiatric or neurological conditions often experience accelerated functional decline despite relatively preserved peripheral health. Preventing, mitigating, and restoring function after episodic neural stress is therefore essential to preserving long-term brain health.

Cognitive resilience as a core determinant of healthspan

Chronological age provides limited insight into functional aging. Peripheral biomarkers, while valuable, capture isolated physiological domains rather than integrated system performance. Cognitive resilience, defined as the brain’s capacity to maintain function, adapt to stress, and recover from perturbation, offers a more comprehensive indicator of longevity potential and has emerged as a target for personalized strategies to preserve brain health across the lifespan [9].

Consider two individuals of identical chronological age and comparable cardiometabolic profiles. One maintains intact executive function, stable sleep architecture, and emotional regulation. The other experiences fragmented sleep, recurrent depressive episodes, and declining attentional control. Despite similar peripheral health, their projected healthspan diverges sharply.

Cognitive resilience encompasses executive function, attentional control, emotional regulation, sleep integrity, and adaptive behavior. Preservation of these domains predicts functional independence, lower morbidity, and reduced mortality risk. If longevity medicine aims to preserve not only survival but functional life, cognitive resilience must be treated as a primary endpoint rather than a secondary outcome.

The brain-first longevity framework

We refer to this model as the Brain-First Longevity Framework (BFLF). Within this framework, preservation and restoration of neural network integrity are positioned as the primary determinants of durable healthspan. Peripheral organ optimization remains necessary but is recognized as downstream and insufficient in isolation (Table 1).

**Table 1:** Peripheral- versus brain-first longevity paradigms This table contrasts prevailing peripheral-first longevity paradigms with the Brain-First Longevity Framework (BFLF) proposed in this article. Peripheral-first approaches prioritize molecular pathways and individual organ preservation as primary drivers of aging, often treating brain-related symptoms as secondary or downstream concerns. In contrast, BFLF positions the central nervous system as the rate-limiting system of longevity, with neural network integrity, cognitive resilience, sleep regulation, and adaptive capacity constraining durable healthspan. The table highlights fundamental differences in aging targets, early indicators of decline, therapeutic priorities, and expected outcomes, illustrating how brain-first approaches reframe longevity from lifespan extension toward preservation of functional independence and resilience.

Domain	Peripheral-First Longevity Paradigm	Brain-First Longevity Framework
Primary aging target	Peripheral organs and molecular pathways	Central nervous system and neural networks
Governing constraint	Organ failure or molecular damage	Neural network integrity and adaptive capacity
Core longevity endpoint	Lifespan extension	Functional healthspan and cognitive resilience
Early aging indicators	Cardiometabolic biomarkers, frailty	Cognitive decline, sleep disruption, affective instability
Role of the brain	Secondary or downstream organ	Primary rate-limiting system
Interpretation of neuropsychiatric symptoms	Quality-of-life issues	Early longevity-limiting pathology
Therapeutic focus	Risk factor reduction and organ preservation	Preservation and restoration of neural network function
Role of episodic neurological events	Often treated as isolated conditions	Cumulative accelerants of brain aging
Therapeutic time horizon	Static or short-term interventions	Longitudinal, adaptive, lifespan-oriented interventions
Expected outcome	Longer survival with variable function	Durable independence, adaptation, and disease resilience

BFLF conceptualizes aging as a progressive reduction in neural adaptive capacity driven by both chronic and episodic stressors. Longevity is achieved not solely by slowing molecular aging but by sustaining network efficiency, flexibility, and resilience over time. This framework reframes brain health from a quality-of-life consideration to a central longevity imperative.

Implications for longevity therapeutics

Recognizing the brain as the rate-limiting organ of longevity necessitates a reevaluation of therapeutic priorities. Neuroprotection alone is insufficient to sustain long-term brain function. Preservation of healthspan requires ongoing maintenance and restoration of neural networks across the lifespan.

BFLF therapeutics can be broadly categorized into neuroprotective, neurorestorative, and neuroadaptive interventions. Neuroprotective strategies aim to reduce acute or chronic neural injury. Neurorestorative approaches seek to recover lost function following network disruption. Neuroadaptive therapies maintain network efficiency through repeated, structured engagement over time.

Design principles for brain longevity therapeutics include longitudinal dosing across decades, adaptive personalization, engagement as a pharmacodynamic variable, and prevention of episodic neurotoxicity as a primary therapeutic objective. Pharmacologic therapies may support neural health, but they cannot substitute for interventions that directly engage and reshape neural circuits.

Testable predictions of a brain-first longevity model

BFLF generates several testable predictions. Individuals with preserved cognitive resilience will demonstrate superior longevity outcomes independent of traditional cardiometabolic risk factors. Interventions that measurably improve neural network efficiency will confer broader systemic benefits than peripheral organ-targeted longevity therapies. Recurrent neuropsychiatric or neurological episodes will predict accelerated biological aging even when peripheral biomarkers remain within normal ranges. Longitudinal brain-targeted interventions will demonstrate nonlinear benefits compared with static lifestyle or pharmacologic approaches.

These predictions position brain-first longevity as a falsifiable and empirically testable model rather than a speculative construct.

Clinical and policy implications

BFLF carries immediate clinical implications. Routine assessment of cognitive function, sleep quality, emotional regulation, and autonomic stability should precede and guide longevity interventions. Early cognitive symptoms, sleep disturbance, affective instability, and recurrent neuropsychiatric episodes should be recognized as early longevity-limiting pathology rather than secondary quality-of-life concerns.

Longevity strategies that fail to prioritize brain function risk extending lifespan without preserving agency, independence, or meaning, resulting in prolonged morbidity rather than durable healthspan. At a policy level, scalable, evidence-based strategies to preserve cognitive resilience will be essential to ensure that an extended lifespan translates into meaningful functional longevity.

Conclusion

Longevity is not limited by how long the body survives, but by how long the brain can sustain coherent function. Peripheral organs may determine the final cause of death, but the brain determines the duration and quality of life that precedes it. Any longevity strategy that does not explicitly preserve and restore brain function will ultimately fail, regardless of how effectively it slows peripheral aging. Recognizing the brain as the rate-limiting organ of longevity is therefore not a conceptual preference, but a biological imperative.
